# Unexpected Hemorrhage: Cough-Induced Rectus Sheath Hematoma in a Pregnant Woman on Aspirin

**DOI:** 10.7759/cureus.99240

**Published:** 2025-12-14

**Authors:** Omar Abusedera, Sara Naser, Danial Lakhani, Awrad J Alkhaldi, Yasmeen Akhtar

**Affiliations:** 1 School of Medicine, Royal College of Surgeons in Ireland - Bahrain, Muharraq, BHR; 2 Obstetrics and Gynaecology, Salmaniya Medical Complex, Manama, BHR

**Keywords:** abdominal pain, conservative management, magnetic resonance imaging, pregnancy, rectus sheath hematoma

## Abstract

Rectus sheath hematoma (RSH) is an uncommon and often underrecognized cause of acute abdominal pain, resulting from rupture of the epigastric vessels or direct muscular injury leading to bleeding within the rectus sheath. Its presentation in pregnancy is particularly rare and can mimic other obstetric or surgical emergencies such as placental abruption, appendicitis, or ovarian torsion, making diagnosis challenging. We report the case of a 34-year-old woman, gravida 3 para 2, at 33 weeks’ gestation, who presented with acute right-sided abdominal pain following coughing episodes. MRI confirmed a right rectus sheath hematoma. She was managed conservatively with analgesia, intravenous fluids, and close observation, resulting in full recovery. This case highlights the importance of considering RSH in the differential diagnosis of abdominal pain during pregnancy, as prompt recognition and conservative management can prevent unnecessary surgical intervention and ensure favorable maternal and fetal outcomes.

## Introduction

Acute abdominal pain in pregnancy can be challenging to diagnose because many conditions may present similarly. One rare but important cause is rectus sheath hematoma (RSH), which can easily be mistaken for more common intra-abdominal emergencies and carries a misdiagnosis rate of 93%. Risk factors include anticoagulant use, bleeding disorders, prior abdominal surgery, laparoscopic injury, subcutaneous injections, or situations that increase intra-abdominal pressure, such as coughing, straining, or pregnancy itself [1].

RSH occurs when blood collects within the sheath of the rectus abdominis muscle, usually due to a vessel rupture or muscle tear [1]. The pain can range from mild discomfort to severe, sharp pain. In pregnancy, the enlarging uterus may worsen symptoms, and large hematomas can compromise maternal circulation, potentially leading to placental hypoperfusion and fetal distress [2].

Although it is a rare condition in pregnancy, incidence rate is estimated at 1.8 cases per 100,000 in the general population [1]. RSH carries significant risks in pregnancy, with reported maternal mortality up to 13% and fetal mortality as high as 50% [3].

Here, we share the case of a 34-year-old woman at 33 weeks’ gestation with a history of chronic cough who developed RSH, focusing on her presentation, investigation, and management.

## Case presentation

A 34-year-old woman, gravida 3 para 2, at 33 weeks’ gestation, presented to the obstetric emergency department at Salmaniya Medical Complex on October 1, 2025, with acute right-sided abdominal pain. The pain had begun three days earlier as mild discomfort in the right lumbar region and had progressively worsened, becoming severe on the day of presentation. The pain followed episodes of coughing but was not associated with nausea, vomiting, vaginal bleeding, or fluid leakage. Fetal movements were perceived normally.

Her medical history was significant for essential hypertension and sickle cell trait. She had been hospitalized in June 2025 for elevated blood pressure and maintained on methyldopa 500 mg three times daily and aspirin 100 mg daily as prophylaxis against preeclampsia. She also took ferrous sulfate 80 mg daily. There was no history of trauma, anticoagulant therapy, or recent invasive procedures.

On examination, she was in mild distress due to pain but hemodynamically stable (blood pressure 105/63 mmHg, pulse 100 bpm, temperature 36.6°C). Abdominal examination revealed a 32- to 33-week gravid uterus, relaxed, with marked tenderness over the right upper and lumbar regions but no guarding or rigidity. Fetal heart tones were reassuring, and urine albumin was negative. Examination did not reveal a palpable abdominal wall mass, indicating a negative Fothergill’s sign. Although Carnett’s sign was not formally assessed at the time of presentation, the localized tenderness without guarding and the absence of peritoneal irritation were consistent with an abdominal wall source of pain such as rectus sheath hematoma.

Given the patient’s gestational age and acute right-sided pain, the initial differential diagnosis included appendicitis, ovarian torsion, degenerating fibroid, and placental abruption, in addition to abdominal wall pathology. These possibilities were considered during the early assessment based on the location and severity of pain, absence of peritoneal signs, and normal fetal status. The combination of severe, localized abdominal wall tenderness with preserved hemodynamic stability and no guarding or rigidity raised early consideration for an abdominal wall pathology rather than intra-abdominal causes. Carnett’s sign could not be reliably assessed due to pregnancy discomfort, but the clinical picture still favored a possible rectus muscle injury.

A transabdominal ultrasound revealed a single, viable fetus in cephalic presentation with normal amniotic fluid and an anterior placenta without retroplacental collection, thus excluding placental abruption. However, a 5×1.5 cm hypoechoic lesion was identified along the right abdominal wall, raising the possibilities of a degenerating fibroid, cyst, hematoma, or ovarian torsion, while appendicitis could not be excluded. Ultrasound images were not archived in the hospital system at the time of the emergency examination. The identification of a localized abdominal wall lesion adjacent to the rectus muscle combined with the patient’s recent coughing episodes shifted clinical suspicion further toward rectus sheath hematoma.

**Figure 1:**
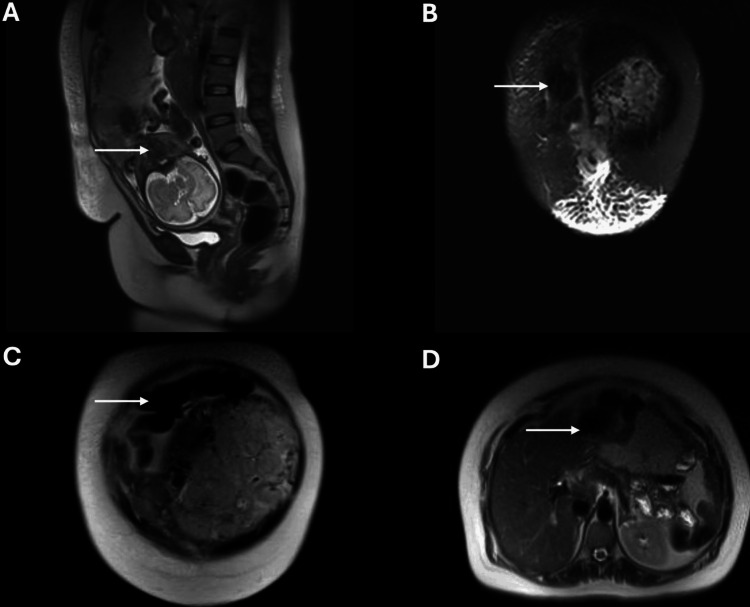
Magnetic resonance imaging (MRI) of the abdomen and pelvis showing a right-sided rectus sheath hematoma in a 33-week pregnant patient. (A) Sagittal T2-weighted image showing the hematoma compressing the anterior abdominal wall and the gravid uterus inferiorly. (B,C) Axial T2-weighted images showing a heterogeneous hyperintense lesion within the right rectus sheath. (D) Axial T1-weighted image demonstrating a hypointense region consistent with an organized hematoma, without signs of active bleeding.

Due to persistent pain and diagnostic uncertainty, a magnetic resonance imaging of the abdomen and pelvis without contrast was requested. Despite technical limitations from the advanced gestation and maternal discomfort, imaging showed a bulky right rectus muscle with heterogeneous signal intensity and overlying subcutaneous edema, features suggestive of a RSH (Figure 1A-D). Laboratory findings were as follows: hemoglobin: 9.4 g/d, white blood cell count: 11.5×10⁹/, Platelets: 293×10⁹/L, fibrinogen: 518 mg/dL. Renal, liver, and electrolyte profiles were within normal limits, urine protein was negative, and tumor markers (cancer antigen 125 (CA-125), cancer antigen 19-9 (CA 19-9), carcinoembryonic antigen (CEA), alpha-fetoprotein (AFP)) were within the expected range for pregnancy.

A multidisciplinary consultation involving obstetrics and general surgery concluded that the findings were most consistent with a RSH. She opted for conservative management and was kept nil per os (NPO) in anticipation of possible intervention if her condition deteriorated. Because the patient remained hemodynamically stable, with a non-expanding hematoma, normal coagulation studies, and no evidence of fetal compromise, conservative management was deemed appropriate. Surgical intervention was not indicated as there were no signs of active bleeding, progressive enlargement, or intraperitoneal extension requiring operative or radiologic intervention.

Repeat laboratory tests obtained during hospitalization on November 25, 2025 showed hemoglobin at 10.4 g/dL, white blood cell count at 9.55×10⁹/L, and platelets at 290×10⁹/L, consistent with stable hematologic parameters. Coagulation testing showed a normal international normalized ratio (INR) and activated partial thromboplastin time (aPTT) ratio, with no evidence of coagulopathy. Electrolytes and renal and liver function remained within normal pregnancy-adjusted limits. Vital signs remained stable throughout management, with no episodes of hypotension, tachycardia, or clinical deterioration. Table 1 shows the laboratory values on admission (October 1, 2025) and at follow-up during hospitalization (November 25, 2025).

**Table 1:** Laboratory results on admission (October 1, 2025) and at follow-up during hospitalization (November 25, 2025), demonstrating stable hematologic, biochemical, and renal parameters throughout conservative management. Reference ranges are provided with pregnancy-adjusted values where applicable. Fibrinogen was not repeated during follow-up due to a clotted sample but was expected to remain within the physiologic pregnancy range.

Parameter	October 1, 2025	November 25, 2025	Reference range
Hemoglobin	9.4 g/dL	10.8 g/dL	11.6–15.0 g/dL
White blood cell count	11.5×10⁹/L	9.55×10⁹/L	3.4–9.6×10⁹/L
Platelets	293×10⁹/L	290×10⁹/L	150–450×10⁹/L
Fibrinogen	518 mg/dL	–	350–600 mg/dL

**Figure 2:**
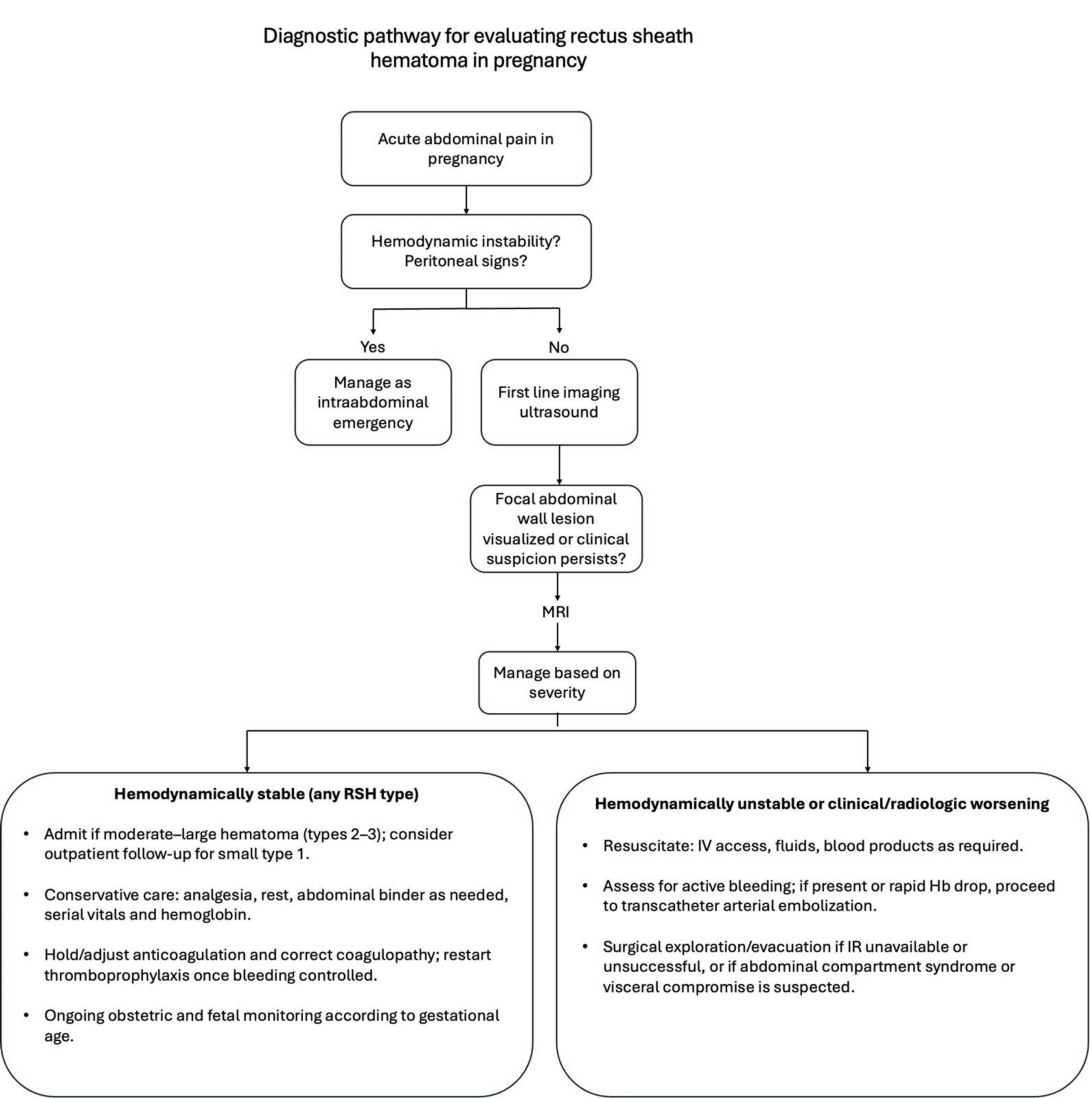
Diagnostic pathway for evaluating rectus sheath hematoma (RSH) in pregnancy

Figure 2 provides a suggested diagnostic pathway summarizing this approach.

## Discussion

RSH is a rare cause of severe abdominal pain, resulting from epigastric vessel rupture or direct muscle injury, causing bleeding into the rectus sheath. It is easily misdiagnosed and only accounts for <2% of acute abdominal pain presentations [4]. Although data are limited, a 1999 study found RSH in 1.8% of patients undergoing ultrasound for acute abdominal pain [5]. In pregnancy, only case reports and series have been published so far and incidence rates have not been well-documented.

The reported risk factors include anticoagulant therapy, trauma, cough, hypertension, increased abdominal tension, female sex, and older age. Among these, anticoagulation remains the most frequently reported cause, accounting for nearly two-thirds of the cases [6,7]. Our patient was not on any such therapy. Instead, she had a background of essential hypertension and reported a recent history of cough, both recognized contributors to vascular rupture. These, together with the mechanical stress of a gravid uterus at 33 weeks, likely precipitated the hematoma formation in her case. Although our patient had sickle cell trait, this condition is generally not associated with increased bleeding risk or spontaneous hematoma formation. SCT rarely causes vaso-occlusive events except under extreme hypoxic or dehydrating stress, and there is no evidence linking SCT to rectus sheath hematoma. Therefore, it is unlikely to have contributed to the hematoma in this case.

The patient was also taking low-dose aspirin as prophylaxis against preeclampsia. Aspirin is known to impair platelet aggregation and has been reported as a contributing factor in cases of spontaneous soft-tissue bleeding, including abdominal wall hematomas [8]. While low-dose aspirin is generally considered safe during pregnancy, it may still slightly increase bleeding tendency in susceptible individuals, particularly when combined with triggers such as coughing or increased abdominal wall tension. In this case, aspirin use may have played a minor contributory role, although it is unlikely to have been the sole precipitating factor.

Signs and symptoms of RSH are usually non-specific. The most frequent symptom is severe, sharp, constant, unilateral, non-radiating abdominal pain, developing acutely or over hours. It occurs in 90% of patients and sometimes worsens with movement or changes in position (Carnett’s sign). A palpable, non-pulsating mass (Fothergill's sign) is another common sign present in 63%-92% of cases and is usually tender [4]. It often occurs in the right lower quadrant due to dominant right-sided activity and anatomic variations below the arcuate line [4,9]. This aligns with our patient’s examination findings. Cullen’s and Grey Turner’s signs can also be present but are not as common [10]. Finally, signs of hypovolemia (hypotension, tachycardia, dizziness), urinary or gastrointestinal disturbances, and features of peritoneal irritation may also occur [4]. The overall clinical presentation is variable and depends on the hematoma’s size, location, and degree of peritoneal involvement.

There are three types of hematomas that can be classified by computer tomography (CT) scan: Type I, which is unilateral and occurs within the rectus muscle; Type II, which can also be bilateral and between the rectus sheath and transversalis fascia; and Type III, which is the most severe, extending into the peritoneum and requiring blood transfusions [11]. Although this classification is based on CT imaging, the MRI findings in our patient showing a unilateral hematoma confined within the rectus muscle without extension into deeper fascial layers are most consistent with a Type I rectus sheath hematoma. This aligns with her stable clinical course and favorable response to conservative management.

Most cases are self-limited and uncomplicated. However, severe bleeding can lead to hemodynamic instability and hypovolemic shock, requiring blood transfusions or surgical intervention. A large hematoma can lead to fetal distress and preterm labor, which is why RSH needs to be taken seriously [3]. Our patient’s hemoglobin on admission was 9.4 g/dL, which is consistent with the mild physiological anemia of pregnancy. The stable repeat value and normal coagulation profile indicated that the hematoma was not actively bleeding, further supporting the appropriateness of conservative management with no blood transfusions needed.

Ultrasound is the first-line investigation for investigating acute abdominal pain and has a sensitivity of 80%-90% for identifying RSH, which typically appears as spindle-shaped on longitudinal scans and as an ovoid mass on transverse and coronal sections. The mass usually appears homogeneous and sonolucent but, in the presence of clot, could appear heterogeneous. CT scan provides more accurate details like location, size, origin, extent and nature of the hematoma and offers better sensitivity and specificity than ultrasound. CT scan usually shows a hyperdense mass, located posterior to the rectus abdominis, with ipsilateral enlargement of the muscle. If the hematoma is chronic, it could appear isodense or even hypodense compared to the surrounding muscle tissue [4]. However, CT scans are avoided during pregnancy due to radiation risks and thus an MRI is used even though lower diagnostic results may be achieved, especially in the first 48 hours, compared with the gold-standard CT scan [3].

Management is usually conservative for uncomplicated presentations with a clear diagnosis and mild symptoms. This involves analgesics, hematoma compression to reduce swelling, ice packs, and bed rest. If hemodynamically unstable with significant blood loss, intravenous fluids and blood transfusion, respectively, must be considered. If a hematoma is expanding, arterial embolization (via vessel coiling) or surgical decompression, performed by ligating the bleeding vessel and removing the hematoma, is the treatment of choice [3].

This case report has a few limitations. First, the ultrasound images obtained during the initial emergency evaluation were not archived in the hospital system, which restricts visual comparison between the preliminary and confirmatory imaging modalities. Second, serial quantitative measurements of the hematoma size were not recorded during hospitalization, limiting the ability to objectively track interval changes prior to discharge. Despite these limitations, the clinical presentation, imaging findings, and symptom trajectory provide a clear and reliable description of rectus sheath hematoma in pregnancy.

## Conclusions

We reported a rare case of RSH in a 34-year-old woman at 33 weeks’ gestation, presenting with acute right-sided abdominal pain. MRI confirmed the diagnosis after ultrasound was inconclusive, and the patient was successfully managed conservatively with full recovery and favorable maternal and fetal outcomes.

RSH should be considered early in the differential diagnosis of acute abdominal pain in pregnancy, particularly when pain is localized, hemodynamic stability is preserved, and peritoneal signs are absent. Based on this case and existing literature, a practical diagnostic pathway can be recommended: ultrasound should remain the first-line imaging modality, given its safety and accessibility; however, when a focal abdominal wall lesion is visualized or clinical suspicion persists despite inconclusive findings, clinicians should proceed promptly to MRI rather than pursuing extensive evaluation for intra-abdominal causes. Early recognition and targeted imaging avoid diagnostic delays, reduce unnecessary surgical exploration, and optimize maternal-fetal outcomes.
